# Sex-specific and regional differences in the prevalence of diagnosed autoimmune diseases in Germany, 2022

**DOI:** 10.1007/s43999-025-00061-5

**Published:** 2025-03-26

**Authors:** Manas K. Akmatov, Claudia Kohring, Frank Pessler, Jakob Holstiege

**Affiliations:** 1 Department of Epidemiology and Health Care Atlas, Central Research Institute of Ambulatory Health Care, Salzufer 8, Berlin, 10587 Germany; 2 TWINCORE Centre for Experimental and Clinical Infection Research, Hannover, Germany; 3 Helmholtz Centre for Infection Research, Braunschweig, Germany

**Keywords:** Autoimmune diseases, Prevalence, Epidemiology, Regional differences, Rheumatoid arthritis, Sex-specific differences, Type 1 diabetes

## Abstract

**Background:**

Research on the epidemiology of autoimmune diseases is impeded due to the rarity of most autoimmune diseases. We aimed to assess the prevalence of diagnosed autoimmune diseases in Germany and examine their sex-specific and regional differences.

**Methods:**

A cross-sectional study using the nationwide ambulatory claims data of females and males of any age with statutory health insurance from 2022 was designed (*N* = 73,241,305). Autoimmune diseases were identified by diagnostic codes of the International Classification of Diseases and Related Health Problems, 10th Revision, German Modification (ICD-10-GM). Regional differences were examined at the level of urban and rural districts (*N* = 401). To control for demographic differences across districts we applied the direct standardization method to calculate sex- and age-standardized prevalences with the German population in 2022 used as a standard population. Furthermore, we calculated prevalence ratios (PR) and 99% confidence intervals (99% CI) to examine sex differences.

**Results:**

Of 73,241,305 insurees (median age, 45; interquartile range, 26–63 years), 6,307,120 had at least one (any) autoimmune disease in 2022, corresponding to a crude prevalence of 8.61% (99% CI: 8.60–8.62%). Of all individuals with autoimmune diseases, 67% were females. The prevalence of single autoimmune diseases varied between 0.008% (pemphigus) and 2.3% (autoimmune thyroiditis). Other autoimmune diseases with a high prevalence were psoriasis (1.9%), rheumatoid arthritis (1.4%), and type 1 diabetes (0.75%). The prevalence was higher in females than males for 25 of the 31 autoimmune diseases with the highest PR observed for autoimmune thyroiditis (PR 5.92; 99% CI: 5.88–5.95), primary biliary cirrhosis (5.60; 5.36–5.84) and systemic lupus erythematosus (5.15; 4.97–5.36). Males were more likely to be diagnosed than females with type 1 diabetes (1.37; 1.36–1.39), ankylosing spondylitis (1.40; 1.39–1.43) and Guillain-Barré syndrome (1.31; 1.27–1.37). The only autoimmune disease without sex difference was myasthenia gravis (1.00; 0.97–1.03). At district level the age- and sex-standardized prevalence of at least one (any) autoimmune disease differed by a factor of nearly 2 between 5.91% and 11.62%. In general, the prevalence was higher in East (former GDR) than West (former FRG) Germany.

**Conclusion:**

Although most autoimmune diseases were rare, when considered as a whole, autoimmune diseases turned out to be more common than previously assumed, with one out of 12 individuals affected in Germany.

**Supplementary Information:**

The online version contains supplementary material available at 10.1007/s43999-025-00061-5.

## Introduction

Research on the epidemiology of autoimmune diseases is impeded due to the rarity of most autoimmune diseases. This results in a paucity of large-scale, epidemiological studies with sufficient sample sizes aiming to assess the prevalence of rare autoimmune diseases in general populations. In fact, previous research on epidemiology of autoimmune diseases in Germany mostly concentrated on single autoimmune diseases such as rheumatoid arthritis [1], multiple sclerosis [2], systemic lupus erythematosus [3] or on a small group of related diseases [4]. Studies examining a wide range of autoimmune diseases using the same study population and with a comparable methodology are lacking. One recent systematic review extracted prevalence estimates for several inflammatory rheumatic diseases in Germany such as rheumatoid arthritis, systemic lupus erythematosus, the Sjogren’s syndrome, ankylosing spondylitis and systemic sclerosis [5]. However, the comparability of these findings is limited due to varying study populations involved and methods used. Nationwide claims data provide a unique opportunity to study the epidemiology of autoimmune diseases in the entirety. The aim of this study was to assess the prevalence of autoimmune diseases in the population of individuals with statutory health insurance (SHI), who make up about 87% of the total population in Germany in 2022 and examine their sex-specific and regional differences.

## Methods

### Study design and population

We designed a cross-sectional study using nationwide ambulatory claims data. In brief, claims data are reported by SHI-authorized physicians for billing purposes and contain outpatient diagnoses coded according to the International Classification of Diseases and Related Health Problems, 10th Revision, German Modification (ICD-10-GM) as well as basic demographic characteristics of insurees (sex, age and region of residence). The study population comprised female and male insurees of any age who consulted a SHI-authorized physician at least once in 2022 (*N* = 73,241,305).

### Case ascertainment

In total, 31 autoimmune diseases were examined in the current study. Diagnostic codes for autoimmune diseases were adopted from [6] and slightly modified (Table 1). A person was defined as having an autoimmune disease if he/she was diagnosed with a corresponding ICD-10 code in one quarter and received a confirmed diagnosis in another quarter in 2022. In addition, both ICD-10 codes need to have the diagnostic modifier “assured”.

**Table 1:** Patients with autoimmune diseases as well as crude and sex- and age-standardized^a^ prevalence, 2022

Affected system according to the ICD-10 chapters	Type of autoimmune disease	Autoimmune diseases	ICD-10-GM code(s)	Number of patients, n	Crude prevalence, %	Standardized prevalence, %
Diseases of the blood	Hemolytic	Pernicious anemia	D51.0	84,986	0.12	0.11
		Autoimmune hemolytic anemia	D59.1	10,134	0.014	0.013
		Idiopathic thrombocytopenic purpura	D69.3	37,969	0.05	0.05
Endocrine diseases	Organ-specific	Thyrotoxicosis	E05.0	301,952	0.41	0.39
		Autoimmune thyroiditis (Hashimoto thyroiditis)	E06.3	1,682,930	2.3	2.2
		Type 1 diabetes	E10	549,984	0.75	0.73
		Primary adrenocortical insufficiency (Addison disease)	E27.1	11,238	0.015	0.015
Neurological diseases	Organ-specific	Multiple sclerosis	G35	265,148	0.36	0.35
		Guillain-Barré syndrome	G61.0	15,622	0.02	0.02
		Myasthenia gravis	G70.0	26,696	0.04	0.03
Diseases of the eye	Organ-specific	Iridocyclitis	H20	61,022	0.08	0.08
Gastrointestinal diseases	Organ-specific	Crohn's disease	K50	255,884	0.35	0.34
		Ulcerative colitis	K51	298,617	0.41	0.40
		Primary biliary cirrhosis	K74.3	29,898	0.04	0.04
		Autoimmune hepatitis	K75.4	28,193	0.04	0.04
		Celiac disease	K90.0	115,752	0.16	0.15
Diseases of the skin and subcutaneous tissue	Organ-specific	Pemphigus	L10	6,060	0.008	0.008
	Systemic	Pemphigoid	L12	14,662	0.02	0.02
	Systemic	Psoriasis	L40	1,357,989	1.9	1.8
	Organ-specific	Alopecia areata	L63	72,860	0.099	0.097
	Organ-specific	Vitiligo	L80	89,422	0.12	0.12
Diseases of the musculoskeletal system and connective tissue	Systemic	Rheumatoid arthritis	M05/M06	994,164	1.4	1.3
	Systemic	Psoriatic arthritis	L40.5/M07.0/M07.1/ M07.2/M07.3	17,999	0.02	0.02
	Systemic	Juvenile arthritis	M08/M09.0	34,560	0.05	0.05
	Systemic	Granulomatosis with polyangiitis	M31.3	13,437	0.02	0.02
	Systemic	Polymyalgia rheumatica and/or giant cell arteritis	M31.5/M31.6/M35.3	306,960	0.42	0.40
	Systemic	Systemic lupus erythematosus	M32.1/M32.8/M32.9	37,8655	0.05	0.04
	Systemic	Dermatomyositis/polymyositis	M33	12,782	0.017	0.017
	Systemic	Systemic sclerosis	M34	35,862	0.05	0.05
	Systemic	Sjogren’s syndrome	M35.0	260,161	0.35	0.33
	Systemic	Ankylosing spondylitis	M45	212,296	0.29	0.28
	**At least one (any) rheumatic disease**			**1,743,393**	**2.38**	
**Total**	**At least one (any) autoimmune disease**			**6.307.120**	**8.61**	**8.46**

^a^Sex- and age-standardized using the direct standardization method. The German population in the year 2022 obtained from the Federal Statistical Office was used as a standard population [7]

### Statistical analysis

We calculated the proportion (i.e. prevalence) of individuals with at least one (any) autoimmune disease and for each of the 31 autoimmune diseases. Furthermore, to control for influences of demographic differences across districts we calculated a sex- and age-standardized prevalence using the direct standardization method. As a standard population we used German population in 2022 obtained from the German Federal Statistical Office [7]. To examine the sex differences in the prevalence of autoimmune diseases, we calculated prevalence ratios (PR) with corresponding 99% confidence intervals (99% CI). The latter – a more conservative estimate over the conventional 95% CI—was selected to increase the level of certainty of the estimated parameters. Data were visualized with the R Foundation for Statistical Computing, version 4.3.1 [8].

## Results

The study population comprised 73,241,305 persons with SHI, of whom 53% were females. The median age of insurees was 45 years (interquartile range, 26–63 years). Of 73,241,305 insurees, 6,307,120 had at least one (any) autoimmune disease in 2022, corresponding to a crude prevalence of 8.61% (99% CI: 8.60–8.62%). The sex- and age-standardized prevalence was slightly lower (8.46%; 99% CI: 8.45–8.47%). Of all individuals with autoimmune diseases, 4,200,581 (67%) were females. The corresponding prevalence of any autoimmune disease was higher in females than males (10.7% vs. 6.2%), a higher prevalence in females was observed in all age groups (Supplementary figure S1). Regarding age, the prevalence increased in both sexes constantly achieving the peak at the age group of 75–79 years in females (17.9%) and 80–84 years in males (13.3%).

**Fig. 1:**
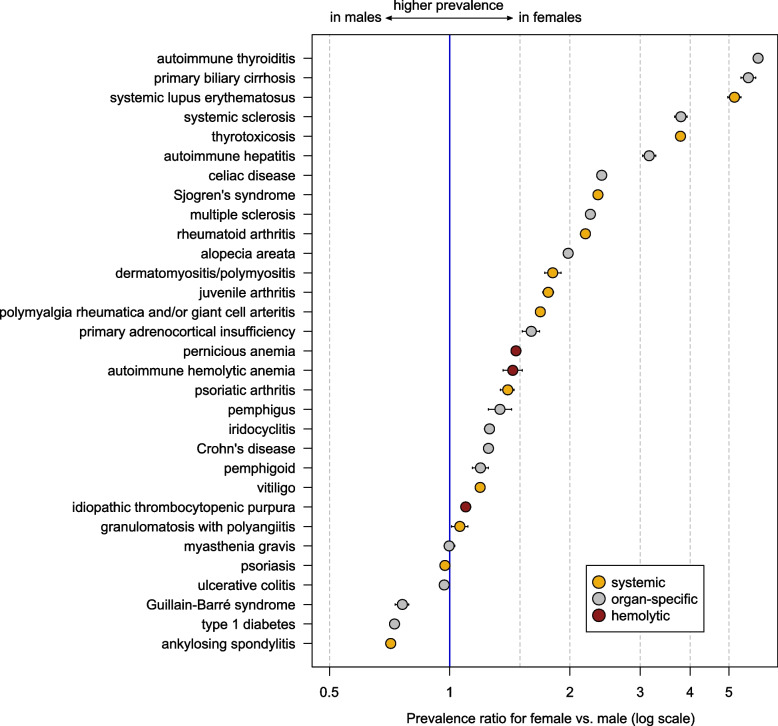
Sex-specific differences in the prevalence of autoimmune diseases, 2022. The values sorted by the magnitude of the prevalence ratio. Whiskers indicate 99% confidence intervals

The autoimmune diseases with the highest prevalence were autoimmune thyroiditis (2.3%), followed by psoriasis (1.9%), rheumatoid arthritis (1.4%), and type 1 diabetes (0.75%) (Table 1). The lowest prevalence was observed for pemphigus (0.008%), followed by autoimmune hemolytic anemia (0.014%), primary adrenocortical insufficiency, aka Addison’s disease (0.015%), dermatomyositis/polymyositis (0.017%), granulomatosis with polyangiitis (0.018%), pemphigoid (0.020%) and Guillain-Barré syndrome (0.021%) (Table 1). The prevalence was higher in females than males for 25 of the 31 autoimmune diseases examined (Fig. 1). The highest female-to-male prevalence ratio (PR) was observed for autoimmune thyroiditis (PR, 5.92; [99% CI], 5.88–5.95), primary biliary cirrhosis (5.60 [5.36–5.84]) and systemic lupus erythematosus (5.15 [4.97–5.36]). Males were more likely to be diagnosed than females with ankylosing spondylitis (male-to-female PR, 1.40; 1.39–1.43), type 1 diabetes (1.37; 1.36–1.39), and Guillain-Barré syndrome (1.31; 1.27–1.37). In addition, a marginal sex effect with a slightly higher prevalence in males than females was observed for ulcerative colitis (1.03 [1.02–1.04]) and psoriasis (1.03 [1.02–1.03]). The only autoimmune disorder without the sex difference was myasthenia gravis (1.00 [0.97–1.03]).

**Fig. 2:**
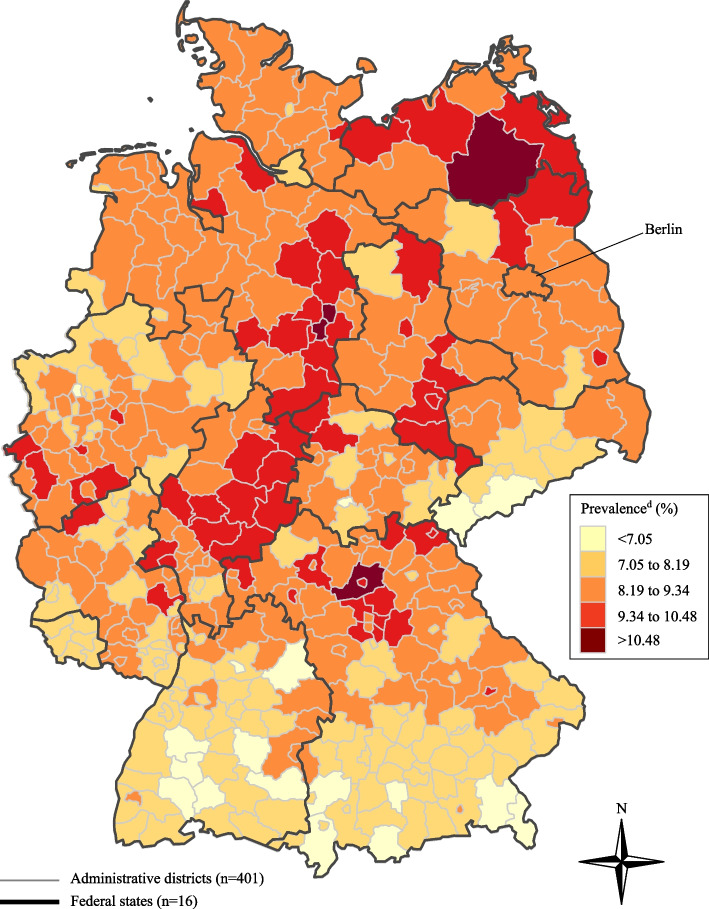
Age- and sex-standardized prevalence^a^ of at least one (any) autoimmune disease^b^ by districts^c^, 2022. ^a^The direct standardization method with the age- and sex-structure of the German population in 2022 as a standard population was used for standardization. German population data were obtained from the Federal Statistical Office in Germany (www.destatis.de). ^b^In total, 31 autoimmune diseases were examined. The list of the autoimmune diseases with the corresponding ICD-10 codes can be found in Table 1. ^c^401 rural and urban districts. ^d^The prevalence was categorized into five equal groups by using equidistant distance

Regionally, we observed differences at several levels. First, the prevalence of at least one (any) autoimmune disease was higher in East (former GDR) than West (former FRG) Germany (Table 2). Second, at the level of federal states the highest prevalence was observed in two eastern federal states, Mecklenburg-Western Pomerania (10.47%) and Saxony-Anhalt (10.26). The lowest prevalence was in Hamburg (7.50%), followed by Baden-Württemberg (7.64%) (Table 2). Third, at district level the age- and sex-standardized prevalence of at least one (any) autoimmune disease differed by a factor of nearly 2 between 5.91% and 11.62%. The highest prevalence (> 10%) was observed in 11 districts (Fig. 2). In addition to the east–west-differences, we observed differences between the North and South parts of Germany with a higher prevalence in northern Germany (Fig. 2).

**Table 2:** Prevalence of at least one (any) autoimmune disease^a^ by sex, region and federal state, 2022

Variables	Prevalence (%)	99% CI
Sex		
Female	10.74	10.73–10.75
Male	6.17	6.16–6.18
Region		
Berlin	8.07	8.03–8.11
East Germany (former GDR)	9.42	9.39–9.44
West Germany (former FRG)	8.47	8.46–8.48
Federal states		
Schleswig-Holstein	9.09	9.04–9.14
Hamburg	7.50	7.44–7.55
Bremen	8.29	8.20–8.38
Lower Saxony	9.20	9.18–9.23
North Rhine-Westphalia	8.59	8.58–8.61
Hesse	8.98	8.94–9.01
Rhineland-Palatinate	8.67	8.63–8.71
Baden-Württemberg	7.64	7.62–7.66
Bavaria	8.24	8.21–8.26
Berlin	8.07	8.03–8.11
Saarland	8.10	8.03–8.18
Mecklenburg-Western Pomerania	10.47	10.40–10.53
Brandenburg	9.65	9.60–9.70
Saxony-Anhalt	10.26	10.21–10.32
Thuringia	9.40	9.34–9.45
Saxony	8.70	8.67–8.74

*CI* Confidence intervals, *FRG* Federal Republic of Germany, *GDR* German Democratic Republic

^a^In total, 31 autoimmune diseases were examined. The list of the autoimmune diseases with the corresponding ICD-10 codes can be found in Table 1

## Discussion

Using the nationwide outpatient claims data comprising information of about 87% of the total population in Germany, we examined the prevalence across a broad spectrum of autoimmune diseases. As expected, the prevalence of most autoimmune diseases was very low with estimates < 1% for 11 disorders and < 0.1% for 17 disorders. Of note, 13 of the 31 autoimmune diseases had a prevalence ≤ 0.05% and thus could be classified as rare diseases. Only three disorders displayed a prevalence between 1 and 3% (rheumatoid arthritis, psoriasis and autoimmune thyroiditis, aka Hashimoto thyroiditis). However, we find that around 6.3 million individuals with SHI were afflicted with an autoimmune disorder, corresponding to a crude prevalence of 8.6%. We are not aware of any other figure (neither observed nor estimated) for the total number of affected individuals in Germany. One systematic review estimated that about 1.4 to 1.9 million individuals in Germany suffer from seven autoimmune diseases, comprising rheumatoid arthritis, polymyalgia rheumatica, juvenile idiopathic arthritis, ankylosing spondylitis, systemic lupus erythematosus, systemic sclerosis, and the Sjogren’s syndrome [5]. It is estimated that 5–10% of the population in developed countries suffer from autoimmune diseases [9]. These estimates vary strongly dependent on the region, involved study populations and the number of examined autoimmune diseases. A study from Denmark reported a prevalence of at least one autoimmune disease of 4% (out of 30 diseases examined also in our study) among 5.5 million individuals in 2006 [6]. A more recent study from the United Kingdom examined the prevalence of 19 autoimmune diseases among 22 million individuals (2017 and 2019) and found that 11% of them were affected by at least one disease [10]. Several studies in Germany assessed the prevalence of individual diseases such as systemic lupus erythematosus [3, 11], myasthenia gravis [12, 13], rheumatoid arthritis [1, 14], ankylosing spondylitis [15, 16], the Sjogren’s syndrome [15, 17], pemphigus and pemphigoid [18], as well as autoimmune hepatitis and primary autoimmune cirrhosis [4]. Although the prevalence estimates are not directly comparable with figures of our study, we observe similar estimates. For example, Mevius et al. [12] observed a prevalence of myasthenia gravis in Germany of 0.042% in 2018 and reported an extrapolated estimate for Germany of 0.039%, which is very similar to our result (0.036%). Two studies reported the prevalence of systemic lupus erythematosus ranging between 0.047% in 2014 [3] and 0.06% in 2019 [11]. We observed a slightly lower prevalence of 0.05%. We did not find any prevalence study for the remaining autoimmune diseases.

### Twenty-five autoimmune diseases are more prevalent in females than males

It is well known that females are more likely to be affected by autoimmune disorders than males due to genetic, epigenetic, hormonal and environmental differences [19, 20]. Recent studies show that the X chromosome is associated with increased susceptibility to autoimmune diseases [21]. In particular, the X chromosome contain several immune-related genes which are associated with autoimmune diseases [19]. On the other side, sex hormones also play an important role in the aetiopathogenesis of autoimmunity/autoimmune diseases [19]. This is supported by the fact, that the risk of autoimmune disease is higher in reproductive age. For example, the female-to-male ratio for systemic lupus erythematosus before puberty is 2:1, while it increases strongly after puberty (9:1) [22]. This finding has also been observed in our study (data not shown). We quantified the sex differences for 31 autoimmune diseases and found a higher prevalence in females for 25 diseases. The strongest sex difference with a PR > 5 was observed for three disorders, autoimmune thyroiditis, primary biliary cirrhosis and systemic lupus erythematosus, followed by thyrotoxicosis, systemic sclerosis and autoimmune hepatitis (PR > 3). Only three disorders were slightly more prevalent in males than females, comprising type 1 diabetes, Guillain-Barré syndrome and ankylosing spondylitis. In addition, very small sex differences were observed for ulcerative colitis (female-to-male PR = 0.97), psoriasis (PR = 0.97) and granulomatosis with polyangiitis (PR = 1.06). The only disorder without sex difference was myasthenia gravis (PR = 1), which has also been observed in another claims-based study involving 3.4 million individuals in Germany [12].

### The prevalence of autoimmune diseases varies regionally

We observed regional differences in the prevalence of autoimmune diseases across various geographical units (e.g. east-west differences or district-level variations). Regional differences may be explained by variations in risk factors such as smoking, obesity, and environmental factors [23, 24]. However, variations in coding behaviour, access to health care, or density of physicians may also contribute to regional differences [25].

### Strengths and limitations

To our knowledge, this is the first study in Germany to examine the prevalence of autoimmune diseases across the spectrum of different autoimmune diseases and quantify sex-specific and regional differences using the same study population and the same methodological procedure to ascertain cases. A further strength of the study is a large study population with nine out of ten residents in Germany included in the dataset. Previous studies using claims data were limited to single regions and were thus less representative for the whole population.

Several limitations of the study need to be mentioned. i) The data were collected for billing purposes and not for epidemiological studies. The resulting limitation is a possible disease misclassification, which may result in both, under- or overestimation of the real prevalence depending on the autoimmune disorder examined. In particular, overestimation based on ICD-10 codes only is known for some diseases such as rheumatoid arthritis [14] or Sjogren’s syndrome [17]. To reduce the risk of misclassification, we applied a conservative case definition, i.e. diagnoses of autoimmune diseases in two different quarters of the year [13, 17]. This may result in underestimation of the real prevalence. Furthermore, there may be regional differences in the documentation of autoimmune diseases. For example, in regions with a high density of physicians (e.g. in urban as compared to rural areas), a more frequent documentation of autoimmune diseases may take place. ii) Case definitions used in the current study were based on ICD-10 classification. We were thus limited to autoimmune diseases with available diagnostic codes and could not examine disorders without specific diagnostic codes (e.g. antiphospholipid syndrome). In addition, a more detailed differentiation for some diseases was not possible (e.g. primary and secondary forms of the Sjogren’s syndrome). iii) The dataset did not contain information for privately insured individuals, which amounts about 10.3% of the German population. This population group has a higher socio-economic status and may thus differ from the SHI population in terms of health and disease risk [26, 27]. Furthermore, about 2.4% of the German population are not insured at all. iv) Finally, the dataset did not contain inpatient information and additional characteristics of the study population such as ethnicity, income or any other sociodemographic data.

## Conclusions

Although most autoimmune diseases were rare, when considered as a whole, autoimmune diseases turned out to be more common than previously assumed, with one out of 12 individuals affected. Future research is needed to better characterize patients with rare autoimmune diseases.

## Supplementary Information

Supplementary Material 1. Supplementary Figure S1. Sex-specific prevalence of at least one (any) autoimmune disease^a^ by age group, 2022. ^a^In total, 31 autoimmune diseases were examined. The list of the autoimmune diseases with the corresponding ICD-10 codes can be found in Table 1.

## Data Availability

The datasets analyzed during the current study are not publicly available due to data protection regulations by the Social Security Code (Sozialgesetzbuch V).
