# Supplementary figures and images for: Sex-specific and regional differences in the prevalence of diagnosed autoimmune diseases in Germany, 2022
Source: Res Health Serv Reg. 2025 Mar 26;4:3. doi: 10.1007/s43999-025-00061-5 (PMC11937456; doi:10.1007/s43999-025-00061-5)

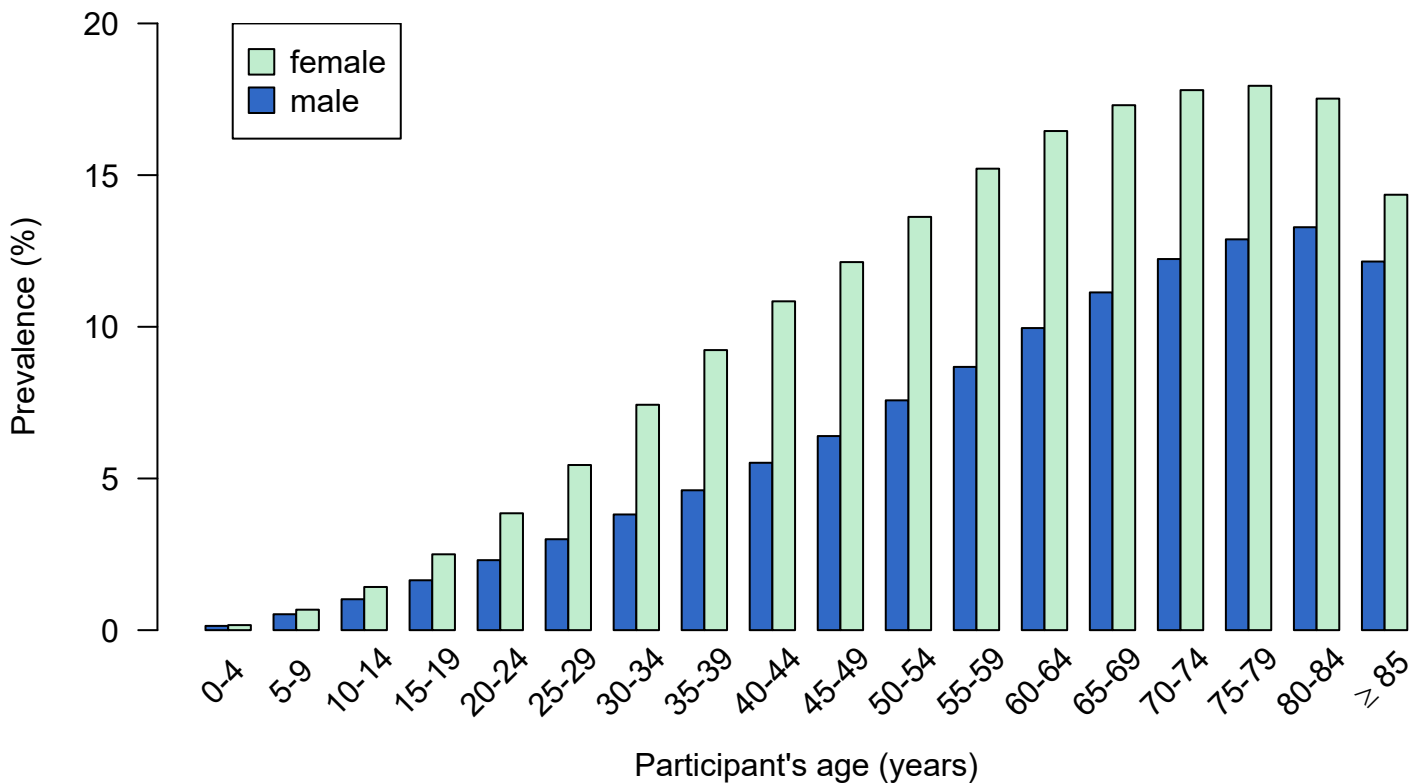

Supplement: Supplementary file 1 — Supplementary Material 1. Supplementary Figure S1. Sex-specific prevalence of at least one (any) autoimmune diseasea by age group, 2022. aIn total, 31 autoimmune diseases were examined. The list of the autoimmune diseases with the corresponding ICD-10 codes can be found in Table 1. [file 43999_2025_61_MOESM1_ESM.pdf]
